# Intranasal Oxytocin Improves Social Behavior in Laboratory Beagle Dogs (*Canis familiaris*) Using a Custom-Made Social Test Battery

**DOI:** 10.3389/fvets.2022.785805

**Published:** 2022-02-24

**Authors:** Borbála Turcsán, Viktor Román, György Lévay, Balázs Lendvai, Rita Kedves, Eszter Petró, József Topál

**Affiliations:** ^1^Institute of Cognitive Neuroscience and Psychology, Research Centre for Natural Sciences, Budapest, Hungary; ^2^Pharmacology and Drug Safety Research, Gedeon Richter Plc., Budapest, Hungary

**Keywords:** oxytocin, dog, social behavior, individual differences, laboratory beagle

## Abstract

For a long time, oxytocin has been thought to have a generally positive effect on social cognition and prosocial behavior; however, recent results suggested that oxytocin has beneficial effects only under certain conditions. The aim of the present study was to explore potential associations between social competence and the effect of intranasal oxytocin on the social behavior of laboratory beagle dogs. We expected oxytocin treatment to have a more pronounced positive effect on dogs with lower baseline performance in a social test battery. Thirty-six adult dogs of both sexes received 32 IU intranasal oxytocin and physiological saline (placebo) treatment in a double-blind, cross-over design, with 17–20 days between the two sessions. Forty minutes after the treatment, dogs participated in a social test battery consisting of eight situations. The situations were carried out within one session and took 20–30 min to complete. Principal component analysis on the coded behaviors identified four components (Willingness to interact, Preference for social contact, Non-aversive response to nonsocial threat, and Non-aversive response to social threat). The subjects' behavior during the placebo condition was used to assess their baseline performance. We found that oxytocin treatment had a differential effect on the behavior depending on the baseline performance of the individuals in all components, but only two treatment × baseline performance interactions remained significant in a less sensitive analysis. In accordance with our hypothesis, oxytocin administration increased dogs' contact seeking and affiliative behaviors toward humans but only for those with low baseline performance. Dogs with low baseline performance also showed significantly more positive (friendly) reactions to social threat after oxytocin administration than after placebo, while for dogs with high baseline performance, oxytocin administration led to a more negative (fearful) reaction. These results indicate that similar to those on humans, the effects of oxytocin on dogs' social behavior are not universally positive but are constrained by individual characteristics and the context. Nevertheless, oxytocin administration has the potential to improve the social behavior of laboratory beagle dogs that are socially less proficient when interacting with humans, which could have both applied and animal welfare implications.

## Introduction

Although the majority of research supports a generally positive effect of oxytocin on social behaviors, this neuropeptide also seems to play critical (and complex) roles also in agonistic or antisocial interactions (1, 2), such as maternal aggression (3). Moreover, a growing number of studies have demonstrated that the effect of oxytocin is not as uniformly beneficial as was previously thought but depends on the characteristics of the individual, on the (perceived) characteristics of the social partner, and on the situation and context itself [see review in Bartz et al. (4)]. In humans, there are numerous studies demonstrating such individual effects, with the best-documented example being the effect of oxytocin in social dilemma games. Oxytocin treatment could promote or actively hinder trust and cooperation in these tasks, depending on the participant's individual traits [e.g., attachment anxiety and avoidance (5, 6)], and on how the participants perceive/categorize their social partner [in-group vs. out-group: (7, 8); trustworthy vs. untrustworthy: (9); known vs. unknown: (10)], or the interaction of these two factors (11). These studies altogether suggest that oxytocin primarily affects anxiously attached, rejection-sensitive participants and promotes higher trust and cooperation toward familiar, trustworthy, or in-group partners, while in less anxious participants and when interacting with unknown, untrustworthy, or out-group partners, it has no or even negative effects (i.e., promotes distrust and less cooperation). These results indicate that oxytocin is not a universal pro-social enhancer but seems to selectively improve social behaviors in individuals who are in need of such enhancement, while others without such need do not benefit from oxytocin. In direct support of this, Bartz et al. (12) showed that oxytocin administration improved performance in an empathic-accuracy task but only in individuals who were less socially proficient during the baseline measurement, whereas it had no effect on more socially proficient individuals.

Contrary to humans, there are only a handful of studies that found similar interactional effects in other species, including dogs. Even though dogs are famous for their unique, human-like social competence (13), their social behavior toward humans is not at all uniform [e.g., Persson et al. (14)]. While plenty of research has shown that oxytocin administration improves the dogs' social approach and human-directed affiliative behaviors [reviewed in Kis et al. (15), Buttner (16)], the question remains whether the effect of oxytocin treatment would be more pronounced in individuals with lower baseline social competence. Two studies, however, lend support to the existence of the differential effect of oxytocin treatment on dogs, depending on their basal levels of oxytocin and the genetic variants they carry on their oxytocin receptor gene (OXTR). More specifically, Romero et al. (17) reported that, after oxytocin nasal intake, social proximity seeking (the tendency to approach their partners) was significantly higher in dogs with low levels of endogenous oxytocin than in dogs with high levels of endogenous oxytocin (measured before intranasal administration). Persson et al. (18) showed that oxytocin treatment increased physical contact seeking with the owners in golden retrievers carrying the AA genotype of the 19131AG OXTR-SNP, but it decreased this behavior in individuals with GG genotype.

Social competence is a multifaceted construct consisting of a range of social–emotional and cognitive skills needed for successful social adaptation. This is one of the central phenomena of social-developmental psychology [see, e.g., Junge et al. (19)], which, however, can also be used to conceptualize the general social ability of an animal during different types and contexts of social interactions (20). Social competence is also a central concept of studies of social cognition in dogs (13) and is often defined as the ability of the dog to exploit available social information to adaptively optimize their social behavioral responses. In dogs, social competence can be measured in the degree to which dogs are effective in their social interactions with other dogs and humans (i.e., their ability to adjust their behavior to meet the demands of different social contexts) when demonstrating cooperative and communicative skills and when to make and sustain social interactions, etc. It is also worth mentioning that different aspects of social competence can be improved through socialization with humans. One reason for the lack of direct empirical evidence regarding the potential moderating effect of the individual's baseline social competence on the effect of oxytocin treatment could be that the majority of studies in this field have been carried out on well-socialized family dogs.

The current study is thus aimed to fill this gap and investigate the effect of oxytocin on dogs with low social competence. It is reasonable to assume that beagles raised with limited human contact under controlled laboratory conditions represent a group with markedly lower social competence than family dogs. However, this group still retains a measure of variability in this regard (21), which makes them ideal subjects to study how baseline social competence alters the effect of oxytocin administration. In this study, we used a double-blind within-subject design; that is, the dogs' responses were investigated in a test battery twice, once after receiving intranasal oxytocin and once after receiving a placebo treatment (in a randomized order). The baseline social competence of each dog was assessed based on their behavioral performance during a placebo treatment. Based on the results of Bartz et al. (12), we hypothesized that the dogs' baseline performance and their treatment would interact, and that oxytocin treatment would have a more pronounced positive effect for dogs with lower baseline performance.

## Materials and Methods

### Subjects

Thirty-seven laboratory beagle dogs participated in the experiment: 15 females and 22 males (none had been neutered). The age of the dogs varied from 2 to 8 years (mean ± SD: 4.75 ± 2.39 years). All dogs were bred by the same commercial breeder who specialized in breeding dogs for laboratory use (WOBE Kft., Budapest, Hungary). The dogs were brought to the research facility where the behavior test took place 1 month before the onset of testing for acclimatization. All dogs were kept indoors, mostly in same-sex pairs in kennels (2 × 2 m), except one male and one female kennel with three dogs in double-sized kennels. The dogs had visual contact with each other. All dogs participated in the same daily care routines including feeding, cleaning, and handling. The dogs were fed once a day at the same time and had an *ad libitum* supply of water, and their interaction with humans was limited to the daily feeding and cleaning of their kennels.

### Procedure

The subjects participated in the test battery twice, with 17–20 days between the two sessions. Two female experimenters (BT and EP) conducted the tests. Half of the dogs were randomly assigned to one experimenter, who played the role of the caretaker for that dog in both test sessions, while the other female experimenter participated in the tests as the “unfamiliar” experimenter. The roles of the experimenters (i.e., caretaker or unfamiliar experimenter) were reversed for the other half of the dogs.

#### Pre-assessment and Familiarization

Two weeks before the first test session took place, the experimenters had visited each kennel to assess whether the dogs were capable of tolerating the presence of an unfamiliar human. One experimenter (the prospective “caretaker”) entered the kennel, crouched down, called both dogs in a friendly manner, petted them, and offered them dry food (the same as their normal daily diet) from her hand. Dogs passed the assessment if they approached the experimenter within 1 min, tolerated petting (did not flee), and accepted the food (*N* = 30 dogs did so).

Dogs that did not meet these criteria (*N* = 7 dogs) participated in a familiarization session (ca. 10 min of social interaction with their assigned caretaker) the next day, with the aim of getting the dogs used to the presence of and physical contact from their caretaker. Familiarization took place in the same room as the behavior test, and the dogs were carried to the testing room by the caretaker. The procedure consisted of four phases.

##### Phase 1 (Passive Interaction, 3 Min)

The caretaker crouched next to the wall and remained passive. Whenever the dog approached her on its own, she gently petted the dog's head and back but did not force the contact and did not initiate interaction.

##### Phase 2 (Active Interaction, 2 Min)

The caretaker stood up, walked to the opposite wall, crouched down, and called the dog.

- If the dog approached her within 10 s, she praised and petted the dog for 20–30 s.
- If the dog did not approach her within 10 s but did not avoid her either, she stepped closer to the dog and gently petted it for 10 s and then stepped back to the wall and waited for 10 s.
- If the dog actively avoided her, she continued to talk to the dog for another 20 s. Whatever the dog's reaction was, after 30 s, the caretaker stood up, walked 3–4 m away, and repeated the above procedure three more times (altogether 4 × 30 s).

At the end of Phase 2, all seven of these dogs were willing to approach the caretaker within 1 min and tolerated physical contact from her.

##### Phase 3 (Food, ~2 Min)

The caretaker showed a piece of dry food (the same as their normal daily diet) to the dog and then put it on the floor and stepped away. If the dog picked up the food within 10 s, she repeated it four more times. If the dog did not eat the food within 10 s, she put down another piece next to it and stepped farther away, repeating this procedure until the dog ate the food (or until 2 min had elapsed). Once the dog ate the food off the floor, the caretaker repeated the same procedure, this time offering food from her hand. At the end of Phase 3, all seven of these dogs were willing to accept food from the caretaker.

##### Phase 4 (Collar and Leash, ~3 Min)

The caretaker put a collar and leash on the floor and a few pieces of dry food next to them. Once the dog sniffed the collar and leash, the caretaker put the collar on the dog, continuously praised and petted the dog for 10 s, and then removed the collar. After ~30 s, she put the collar back on the dog, attached the leash (but left it lying on the floor), continuously praised and petted the dog for 10 s, and then removed the leash. After ~30 s she put the leash back on and walked around the room with the dog on the leash, continuously calling and praising the dog.

#### Oxytocin/Placebo Treatment

The same two female experimenters (BT and EP) who carried out the behavior tests performed the drug treatments, and both were trained in these tasks. The caretaker held the dog while the experimenter administered the dose. Both experimenters were blind as to which treatment the dogs received. Dogs received a single intranasal dose of 32 IU (8 puffs in total, with a half dose administered to each nostril) of oxytocin (Syntocinon, Novartis, Basel, Switzerland) and placebo (physiological saline) treatments in a repeated-measures design. Intranasal administration procedure was unfamiliar to the dogs; thus, some dogs resisted the administration. If a puff was clearly missed (i.e., the dog moved its head right before the experimenter administered the puff), it was repeated. One dog (a 2.1-year-old female) actively and strongly resisted the intranasal administration and thus was excluded from the study. The order of the treatments was balanced between the remaining dogs: *N* = 18 dogs (10 males, 8 females, mean age 5.22 years) received oxytocin before the first test session, and placebo before the second test session; *N* = 18 dogs (12 males, 6 females, mean age 4.43 years) received the treatments in the reversed order. Intranasal administration of oxytocin or placebo was followed by an incubation period (22) spent in the kennels of 35–45 min. After the waiting period, the dog was removed from the kennel and transported to the testing room by the caretaker.

#### Social Test Battery

The test battery consisted of a warm-up phase and 8 tests, and it took ~20 to 30 min to complete. The tests were carried out on the same day and in the same order for all subjects. In some of the tests, the dogs remained leashed by default. However, since the dogs were not familiar with wearing a leash, if the dog showed strong resistance to the leash at any point of the test (i.e., constantly struggled, tried to escape, or displayed freezing behavior) and the caretaker could not calm the dog, she removed the leash and the test continued without it.

##### Warm-Up

The purpose of this phase was to familiarize the subject with the testing environment. The caretaker (hereafter C) carried the dog to the testing room (3 × 5 m) and put it down, and then she sat down on the floor next to the wall. There were objects (including a cardboard box, bags, newspapers, plastic bottles, and a tennis ball) placed around the room. The dog could move freely, while C remained passive in her position. If the dog approached C and initiated the interaction, C briefly petted the dog but otherwise ignored it. The warm-up was terminated after 3 min if the dog explored at least two objects (i.e., spent at least 5 s sniffing or manipulating them) and also approached C at least once during this period. If the dog did not meet these criteria, the warm-up continued until the dog did so or for a maximum of 10 min.

##### Test 1. Interaction With the Caretaker

The aim of this test was to assess how much the dog tolerates/seeks positive interaction with C. The dog was unleashed during the test. The test consisted of 4 trials; each trial was 30 s long.

In the first trial, C stood up and approached the dog in a normal upright (walking) posture and at a normal speed of walk while talking to it in a friendly manner. If the dog was friendly or passive (i.e., showed no avoidance or aggression), C petted the dog and talked to it until the end of the trial (30 s). If the dog moved away, C waited for 15 s and then tried to approach the dog again. If the dog did not move away this time, C petted the dog, and if the dog moved away again, C waited passively until the end of the trial. At the end of the trial, C stepped away from the dog (3–4 steps). In the second trial, C crouched down and called the dog in a friendly manner. If the dog approached her, she petted the dog and talked to it until the end of the trial (30 s). If the dog did not approach her, C waited for 15 s and then approached the dog and petted it. If the dog moved away, C waited passively until the end of the trial. The third and fourth trials were identical to the second one.

##### Test 2. Food Motivation

The aim of this test was to assess the dogs' preference for different food rewards (dry food or sausage) in a social context. The dry food was familiar to the dogs, but the sausage was not (at least during the first food motivation test session). C put a piece of dry food and a piece of sausage on the floor and verbally encouraged the dog to eat them (for 20 s). Then C offered the same food types to the dog from her hand and encouraged the dog to eat them. If the dog ate only the sausage or both food types, the sausage was used in further tests, and if the dog chose only the dry food or none of the foods, dry food was used.

##### Test 3. Greeting by an Unfamiliar Experimenter

The aim of this test was to assess how much the dog tolerates/seeks positive interaction with the unfamiliar experimenter. It was based on the procedure of Turcsán et al. (21). The test consisted of 3 trials. The dog was held on a loose leash (if it tolerated being leashed) by C, who was standing next to it. In the first trial, the unfamiliar experimenter (hereafter E) approached the dog at ~1.5 m and called the dog for 15 s. If the dog stepped toward E, she petted the dog for 7–8 s. If the dog did not approach E, E stepped closer and tried to pet the dog. If the dog showed active avoidance, E talked to the dog for 7–8 s. At the end of the trial, E stepped away from the dog (2–3 steps). The second and third trials were similar to the first, except that E did not approach the dog at the beginning of the trial; she just called it from her location.

##### Test 4. Training for Eye Contact

The aim of this test was to assess how much the dog tolerates/seeks eye contact with C. It was based on the procedure of Wallis et al. (23). The dog was unleashed during the test. The test consisted of a pretraining phase and a test phase.

*Pretraining Phase*. C sat down on the floor next to the wall, called the dog, and offered a piece of food. Then she tried to make eye contact with the dog (by talking in a high-pitched voice, clapping, whistling, etc.). When the dog established eye contact, C praised the dog verbally and gave the food to the dog (if the dog refused to eat, C used only verbal praise). This procedure was repeated once more. C had 3 min to establish eye contact with the dog twice. If C succeeded, the test continued with the test phase; otherwise, the test was terminated.

*Test Phase*. C sat passively by the wall, avoiding any noise or sudden movement that would attract the dog's attention, and watched the dog for 3 min. Every time the dog established eye contact with her on its own, C rewarded the dog (with food and/or verbal praise), and then she continued to passively watch the dog.

##### Test 5. Potentially Threatening Moving Object

The aim of this test was to assess how the dog reacts to the approach of an apparently self-moving object. The object was a remote-controlled toy car (30 cm long, 15 cm wide, and 8 cm high) with a 50 × 30 × 15 cm cardboard box placed over it. The test consisted of two trials. In the first trial, the dog and C were standing at one end of the room. The dog was held on a loose leash. If the dog did not tolerate the leash (i.e., struggled, tried to escape, or froze and did not move), C removed the leash and gently held the dog's body until the test started. E placed the object at the opposite end of the room (~5 m from the dog) and used a remote control to direct the object toward the dog, moving it slowly and haltingly (moving ~1.5 m, then stopping for a few seconds, and then moving again). The approach was terminated if (1) the dog showed active avoidance (retreated to the wall or moved behind C); (2) the dog approached the object; or (3) the object moved within 1 m of the dog. After the approach was terminated, C unleashed the dog (if it was on a leash), carried the object back to its starting point, and then took the dog back to its starting point. The second trial was identical to the first one, except that when the object started moving toward the dog, C stepped ~2 m away from the dog. When the trial ended—either because the dog showed avoidance/approach or because the object moved within 1 m to the dog—C unleashed the dog (if it was on a leash), went to the object, called the dog in a friendly manner (for a maximum of 30 s), and encouraged it to approach/interact with the object.

##### Test 6. Directional Gesturing

The aim of this test was to assess the dog's ability to rely on human directional signals (including pointing and gazing) when choosing between two objects. It was based on the procedure of Hernádi et al. (24). The test consisted of a pretraining phase and a test phase with three trials.

*Pretraining Phase*. C gently held the dog's body. E kneeled on the floor ~1.5 m from the dog and put a plastic plate with a piece of food 20–40 cm in front of the dog. The dog was allowed to eat the food. If the dog refused to eat it, both C and E encouraged the dog until it approached and (at least) sniffed the food/plate. Then they returned to their starting positions.

*Test Phase*. In the first trial, E placed two identical plastic plates on the floor ~1.8 m apart, both containing a piece of food. E attracted the dog's attention (by talking in a high-pitched voice, clapping, whistling, etc.), and once the dog looked at her, E pointed (sustained pointing) and gazed at one of the plates and then looked back at the dog. Then C released the dog. If the dog approached one of the plates at <10 cm, E picked up the other plate. If the dog did not approach any of the plates, E waited for 15 s and then picked up both plates. The second and third trials were similar to the first one, except for the cues given by E. In the second trial, E used momentary pointing, E pointed and gazed at one of the plates (the opposite one to the first trial) for 2–3 s, and then lowered her arm and looked back at the dog before it was released. In the third trial, E did not use pointing but only gazed at one of the plates (the same as in the first trial) for 2–3 s and then looked back at the dog before it was released. The side of the cued plate in the first trial was balanced between the dogs.

##### Test 7. Emotion Recognition

The aim of this test was to assess the dog's ability to rely on the human emotional expressions of joy and fear when choosing between two objects. It was based on the procedure of Turcsán et al. (25). The test consisted of two trials. In the first trial, C held the dog's collar or the dog's body, and E crouched down ~1.5 m from the dog and placed two objects of similar size (a yellow wooden toy and a red plastic cup) on the floor ~1.8 m apart. E stepped to one of the objects, picked it up, and showed the assigned emotional expression (joy or fear) for ~5 s. She expressed the emotion with facial expression, verbal exclamations, and body language and looked at the dog at least once during this time. Then E put down the object, went to the other object, and performed the other emotional expression. When E took up her original standing position (halfway between the two objects), C released the dog. If the dog made a choice (approached one of the objects within 10 cm), E picked up the other object. If the dog did not make a choice, E waited for 15 s and then picked up both objects. The second trial was similar to the first one, except that E demonstrated the two emotions in the reverse order. The objects' location (right or left side), their assigned emotions, and the emotional expression displayed first by E were balanced between the dogs.

##### Test 8. Threatening Approach

The aim of this test was to assess how the dogs respond to a threatening approach by the experimenter. It was based on the procedure of Vas et al. (26). The dog and C were standing at one end of the room, and the dog was held on a loose leash. E stood ~5 m from the dog and called the dog's attention. When the dog looked at her, E started to slowly approach the dog with a slightly bent upper body, staring steadily into the eyes of the dog and without any verbal communication. If the dog interrupted the eye contact with E, she tried to attract the dog's attention by making some noise (coughing or tapping the ground with her foot) and then continued the approach. The approach was terminated if (1) the dog showed active avoidance (retreated to the wall or moved behind C), (2) the dog approached E within arm's reach, or (3) E reached the dog. After the approach was terminated, C unleashed the dog, and E went back to her starting point, crouched down, called the dog in a friendly manner, and petted the dog to resolve the situation.

#### Behavior Coding

All tests were videotaped and analyzed using Solomon Coder (beta 190802 by András Péter, *http://solomoncoder.com/*). Altogether, 28 variables (20 scores, 1 frequency, 7 latencies) were coded in the 8 tests of the battery (Table 1). Neither the coders nor the experimenters had any information about the treatment that the dog received. Note that the number of coded variables was high relative to the number of dogs investigated, and the range and variance of the continuous variables (frequency and latency) were markedly different compared to the score-type variables. We therefore reduced the number of variables and homogenized the range of the different variable types using the following steps.

**Table 1:** Definition of the variables coded in the tests and their inter-observer reliability [Cohen's kappa or intraclass correlation (ICC)].

**Subtest, variable**	**Definition**	**Data processing**
**1. Interaction with the caretaker**		
Reaction to petting	When C tried to pet the dog, the dog: 0, avoided contact (turned or moved away); 1, passively tolerated contact (no sign of contact seeking or avoidance); 2, showed a little contact seeking (shortly sniffed C, kept eye contact); 3, actively sought contact with C (cuddle up, lick, and climb in lap). If the dog behaved differently at the beginning vs. the end of the trial, the mean of the scores assigned to the two behaviors was given.	Coded separately for the four trials. For the analysis, the mean of the four trials was calculated. ICC = 0.927, *F*_14,14_ = 13.792, *p* < 0.001
Latency of approach	From the moment C stepped outside the dog's reach until the dog got within arms' reach of C. If the dog never stepped out of reach, the latency was 0; if the dog did not approach C, the maximum (15 s) was given.	Coded separately for the three approaches. For the analysis, the raw latency was recoded into categories based on its histogram: 0, 0–0.9 s; 1, 1–2.3 s; 2, 2.4–4.5 s; 3, 4.5–9 s; 4, 9–13 s; 5, 13–15 s. Then the mean score of the three approaches was calculated. ICC = 0.816, *F*_14,14_ = 9.863, *p* < 0.001
**2. Food motivation and 6. Directional gesturing**		
Accept food	If the dog ate food (1) or not (0).	Coded separately for the two tests. For the analysis, the two variables were summed. Cohen's kappa = 0.694, *N* = 15, *p* < 0.001
**3. Greeting by an unfamiliar experimenter**		
Reaction to petting	When E tried to pet the dog, the dog: 0, avoided contact (turned or moved away); 1, passively tolerated contact (no sign of contact seeking or avoidance); 2, showed a little contact seeking (shortly sniffed E, kept eye contact); 3, actively sought contact with E (cuddle up, lick, and climb in lap). If the dog behaved differently at the beginning vs. the end of the trial, the mean of the scores assigned to the two behaviors was given.	Coded separately for the three trials. For the analysis, the mean of the three trials was calculated. ICC = 0.683, *F*_14,14_ = 3.020, *p* = 0.024
Latency of approach	From the moment E called the dog/stepped outside the dog's reach until the dog got within arms' reach of E. If the dog approached E before her call or never stepped out of reach, the latency was 0; if the dog did not approach E, the maximum (15 s) was given.	Coded separately for the three trials. For the analysis, the raw latency data were recoded into categories based on its histogram: 0, 0–0.9 s; 1, 1–2.3 s; 2, 2.4–4.5 s; 3, 4.5–9 s; 4, 9–13 s; 5, 13–15 s. Then the mean score of the three trials was calculated. ICC = 0.973, *F*_14,14_ = 47.054, *p* < 0.001
**4. Training for eye contact**		
Frequency of eye contacts	The number of eye contacts the dog established during the test phase (3 min). If the dog did not pass the pretraining phase, 0 (the minimum) was given.	For the analysis, the raw frequency data were recoded into categories based on its histogram: 0, 0–1; 1, 2–10; 2, 11–21; 3, >21. Cohen's kappa = 1.000, *N* = 15, *p* < 0.001
**5. Potentially threatening moving object**		
Type of reaction	The object stopped because the dog: 0, moved away, in the opposite direction as C; 1, moved behind C; 2, was passive; 3, approached the object.	Coded separately for the two trials. For the analysis, the mean of the two trials was calculated. ICC = 0.997, *F*_14,14_ = 341.714, *p* < 0.001
Distance from object	How far the object was from the dog when it stopped: Score 0, ≥4 m; Score 1, ≥2 and <4 m; Score 2, ≥1 and <2 m; Score 3, <1 m.	Coded separately for the two trials. For the analysis, the mean of the two trials was calculated. ICC = 0.995, *F*_14,14_ = 200.714, *p* < 0.001
Latency of sniffing	From the moment C called the dog to the object until the dog approached it at <10 cm. If the dog approached the object on its own while it was still moving, the latency was 0; if the dog did not approach the object at all, the maximum (30 s) was given.	For the analysis, the raw latency data were recoded into categories based on its histogram: 0, 0 s; 1, 1–5 s; 2, 5–20 s; 3, 20–30 s. Cohen's kappa = 1.000, *N* = 15, *p* < 0.001
**6. Directional gesturing**		
Choice	The plate the dog approached at <10 cm: 0, none; 1, any plate.	Coded separately for the three trials. For the analysis, the number of valid choices out of three was calculated. Cohen's kappa = 1.000, *N* = 15, *p* < 0.001
**7. Emotion recognition**		
Choice	The object the dog approached at <10 cm: 0, none; 1, any object.	Coded separately for the two trials. For the analysis, the number of valid choices out of two was calculated. Cohen's kappa = 1.000, *N* = 15, *p* < 0.001
**8. Threatening approach**		
Type of reaction	The dog's final reaction (when the test was terminated): 0, active avoidance (moved away or behind C); 1, passive (no movement toward or away from E); 2, ambivalent (few hesitant steps toward/away from E, may show tail wagging); 3, friendly/appeasing (approached E).	Remained the same for the analysis. Cohen's kappa = 1.000, *N* = 15, *p* < 0.001
Distance from E	How far E was from the dog when she terminated the approach: Score 0, >2 m; Score 1, 1–2 m; Score 2, <1 m; Score 3, the dog approached E.	Remained the same for the analysis. Cohen's kappa = 1.000, *N* = 15, *p* < 0.001

*E, experimenter; C, caretaker*.

First, the continuous variables (frequency and latencies) were recoded into 4 to 6 categories to match the range of the score variables and also to control for extreme values. The threshold values of the categories were decided based on the variables' histograms. Second, for the tests that included repeated trials (Interaction with caretaker, Greeting, and Potentially threatening moving object), we calculated the mean of the trials for each variable coded in that test. Third, in the cases of the Directional gesturing and Emotion recognition tests, the number of dogs that did not make a choice in any given trial was too high (ranging from 35.6 to 58.9%) to assess the dogs' ability to follow directional gestures or emotional cue. Thus, for these two tests, we analyzed the total number of valid choices the dog made. Fourth, we also created a composite score from the two variables assessing the dogs' willingness to eat food (measured in the Food motivation and Directional gesturing tests) by taking the sum of these scores. The final set contained 13 variables; their descriptions can be found in Table 1. Inter-observer agreements for all variables were assessed by double coding *N* = 15 dogs (41.6% of the whole sample) by two independent coders. The inter-observer reliability, assessed by Cohen's kappa or intraclass correlation coefficient (ICC; two-way mixed model, absolute agreement) of all variables, was good or excellent (Table 1).

### Statistical Analysis

The 13 variables coded in the first test session were subjected to principal component analysis (PCA) with Varimax rotation. We used the eigenvalue > 1 rule (27) to determine the number of components retained, and variables that failed to load > 0.5 on any component were excluded in a stepwise manner (28). Cronbach's α was calculated to assess the internal consistency of the resulting components. To calculate the component scores for each dog, we standardized the variables using z-transformation and then calculated the mean of the variables loading with at least 0.5 on a given component (variables that loaded negatively on a component were first multiplied by −1).

We used the component scores of the dog during the placebo treatment condition as a measure of their baseline social competence.

The component scores were used as dependent variables in generalized estimating equation models using restricted maximum likelihood estimation. Dog ID was set as a random factor. For fixed effects, we included the *Treatment* (oxytocin and placebo), the *Test session* (1st/2nd), and the *Sex* (male/female) of the dogs. The *Baseline performance* of that component (the corresponding component score of the dog during the placebo treatment) was entered as a covariate. Two-way interactions between treatment and the other variables were included in the models. Non-significant effects were removed from the model sequentially, in the order of their decreasing significance, starting with the interactions; non-significant main effects were removed only if they had no interaction left in the model (29). Regarding *Baseline performance*, we expected a significant interaction with treatment, which would indicate that oxytocin has a differential effect on the behavior of dogs with high and low social competence. If it was significant, to interpret the interaction, we split the *Baseline performance* at its median to create high and low groups and re-ran the model with this categorical variable. We used sequential Bonferroni correction to adjust the *p*-values of the *post-hoc* tests for multiple comparisons. If this interaction was not significant and was removed from the model, the main effect of *Baseline performance* was also removed, because—being part of the component score itself—this variable on its own was redundant with the dependent variable. All statistical tests were carried out using SPSS v.22.0.

## Results

### Principal Component Analysis

The 13 variables were grouped into four components (Table 2), which together explained a relatively high proportion (76.7%) of the total variance. The first principal component included variables from the Directional gesturing, Emotion recognition, Food motivation, and Training for eye contact tests. Higher scores on this component indicate the dogs' willingness to accept food from a human and to make a choice in response to human directional gestures and emotional displays, as well as their increased tendency to make eye contact with the experimenter. Consequently, this component is referred to as “Willingness to interact.”

**Table 2:** Results of the principal component analysis.

	**Principal components**			
**Variable (** ***test*** **)**	**Willingness** **to interact**	**Preference** **for social** **contact**	**Non-aversive** **response to** **non-social** **threat**	**Non-aversive** **response to** **social threat**
Number of valid choices *(Directional gesturing—test 6)*	**0.913**	0.051	0.200	0.104
Accept food *(Directional gesturing + Food motivation—tests 2 and 6)*	**0.851**	0.164	0.166	−0.036
Number of valid choices *(Emotion recognition—test 7)*	**0.846**	0.014	0.049	0.225
Number of eye contacts *(Training for eye contact—test 4)*	**0.695**	−0.016	0.227	0.031
Reaction to petting *(Interaction with C—test 1)*	−0.137	**0.905**	0.010	0.064
Reaction to petting *(Greeting by E—test 3)*	0.051	**0.890**	0.079	0.068
Latency of approach *(Interaction with C—test 1)*	−0.068	**−0.815**	0.041	0.046
Latency of approach *(Greeting by E—test 3)*	−0.260	**−0.719**	0.061	−0.232
Distance from object *(Potentially threatening moving object—test 5)*	0.066	−0.087	**0.911**	0.040
Type of reaction *(Potentially threatening moving object—test 5)*	0.285	−0.049	**0.856**	0.105
Latency of sniffing *(Potentially threatening moving object—test 5)*	−0.329	−0.182	**−0.697**	−0.245
Type of reaction *(Threatening approach—test 8)*	0.087	0.170	0.129	**0.893**
Distance from E *(Threatening approach—test 8)*	0.118	0.027	0.133	**0.891**
Eigenvalue	4.282	2.788	1.593	1.308
Explained variance	32.940	21.443	12.252	10.060
Cronbach's α	0.861	0.806	0.816	0.819

*Loadings > 0.5 are in boldface*.

*E, experimenter; C, caretaker*.

The second component contained variables from the Interaction with the caretaker and Greeting by an unfamiliar experimenter tests. Higher scores on this component correspond to a more positive reaction to both familiar and unfamiliar humans (i.e., increased contact seeking toward both C and E, and more positive responses upon being petted by both C and E). Based on these, the second principal component is referred to as “Preference for social contact.”

The third component, labeled as “Non-aversive response to non-social threat,” contained variables only from the Potentially threatening moving object situation. A high score on this component indicates a more positive (less fearful) reaction to the approaching unfamiliar object.

The fourth principal component, labeled as “Non-aversive response to social threat,” included variables from the Threatening approach test. A high score on this component is associated with a more positive (less fearful) response to the threatening approach of the experimenter. Cronbach's alpha values were high (>0.8) for all principal components (Table 2), indicating a good degree of internal consistency.

### The Effect of Familiarization, Age, and Sex on the Baseline Social Competence of the Dogs

Seven dogs that initially did not tolerate the presence of physical contact from their prospective caretaker participated in an additional familiarization procedure, with the aim to lower their (social) fear to a level where they could be tested in the battery without causing severe stress for the individuals. Given that the familiarization took place in the same room and utilized a procedure that was similar to later tests, we cannot exclude the possibility that the familiarization may have been more successful than intended, increasing the baseline social competence of these individuals from the lower end to the higher end of the spectrum. Before integrating these dogs together with the rest for later analyses, we first investigated if their baseline social competence was higher than that of the rest of the dogs.

**Figure 1:**
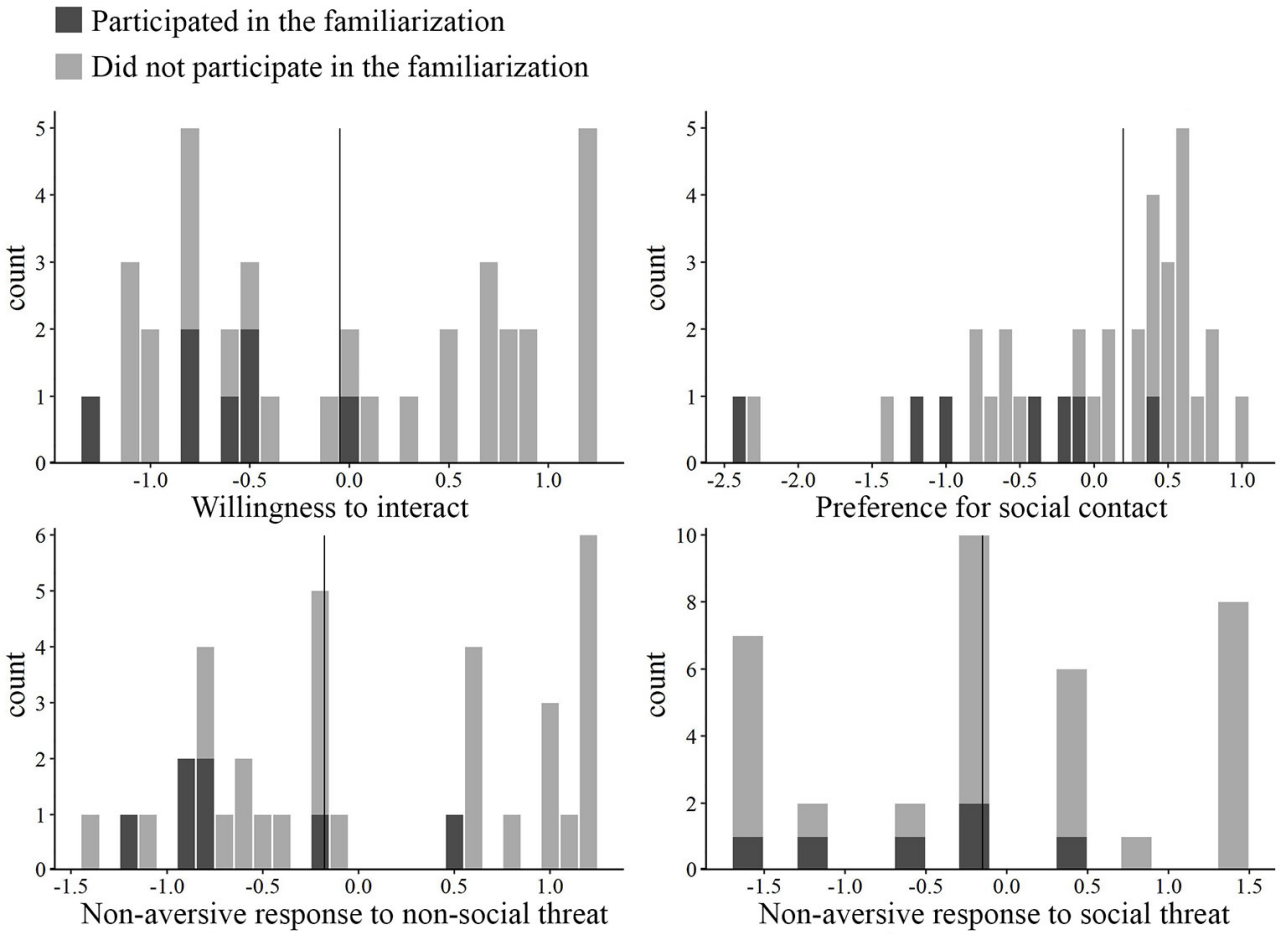
Histograms of the four components during the placebo treatment condition. Vertical lines represent the median that was used as the threshold for dividing the dogs into high and low groups (*N* = 18 in all groups). Dogs that participated in the familiarization are marked with dark gray.

We found that these dogs (*N* = 7) had lower scores in the placebo condition (baseline social competence, Figure 1) as compared to the rest of the dogs (*N* = 29) in all but one component (Mann–Whitney U test, Willingness to interact: z = 1.963, *p* = 0.049; Preference for social contact: z = 2.378, *p* = 0.016; Non-aversive response to non-social threat: z = 2.546, *p* = 0.009). In the case of Non-aversive response to social threat, there was no significant difference between these 7 and the rest of the dogs (z = 0.609, *p* = 0.557). Thus, the results indicated that the baseline social competence of these dogs was still lower or at the same level as the rest of the dogs, which justified pooling the data together for further analyses.

The dogs' component scores during the placebo condition did not correlate with the dogs' age (Spearman correlation, *N* = 36, highest |ρ| = 0.095, *p* > 0.449 for all) and did not significantly differ between males and females (Mann–Whitney *U*-test, *N* = 36, *p* > 0.133 for all).

### Main and Interaction Effects of Treatment, Test Session, Sex, and Baseline Performance

**Figure 2:**
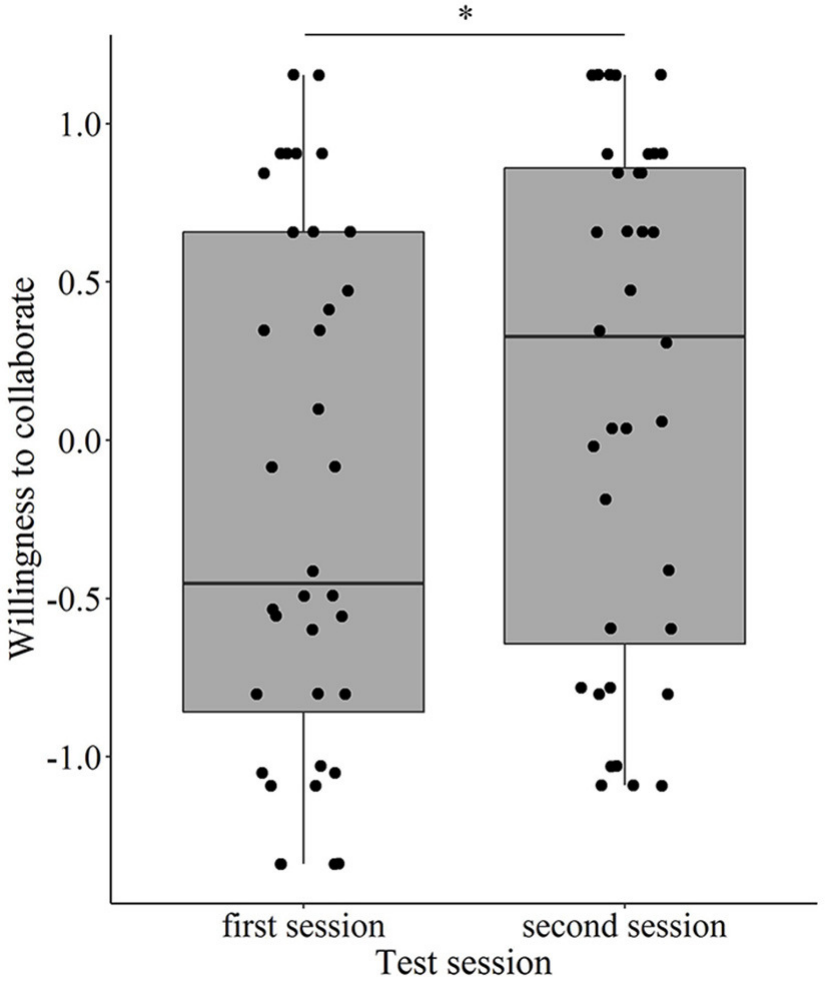
The effect of repeated testing on the dogs' Willingness to interact with human partners (^*^p < 0.001).

Regarding the Willingness to interact component, we found a significant interaction between *Treatment* and *Test session* (χ^2^ = 17.292, *p* < 0.001), and also between *Treatment* and *Baseline performance* (χ^2^ = 5.279, *p* = 0.022). However, when we divided the dogs into low and high groups by the median of the baseline performance and entered this categorical variable in the model to interpret this latter interaction, it was no longer significant (χ^2^ = 2.214, *p* = 0.137). *Post-hoc* pairwise comparisons also did not show a significant difference in the oxytocin treatment between the low and high groups. When this interaction (and consequently, the categorical baseline performance variable) was removed from the model, the *Treatment* × *Test session* interaction also became non-significant (χ^2^ = 0.005, *p* = 0.942), and the final model contained only the main effect of *Test session* (1st/2nd; χ^2^ = 12.562, *p* < 0.001). The dogs showed a higher motivation to engage in the two-way object-choice tasks, were more willing to accept food from the experimenter, and engaged in more eye contact with her during the second (repeated) test session than during the first occasion (Figure 2).

**Figure 3:**
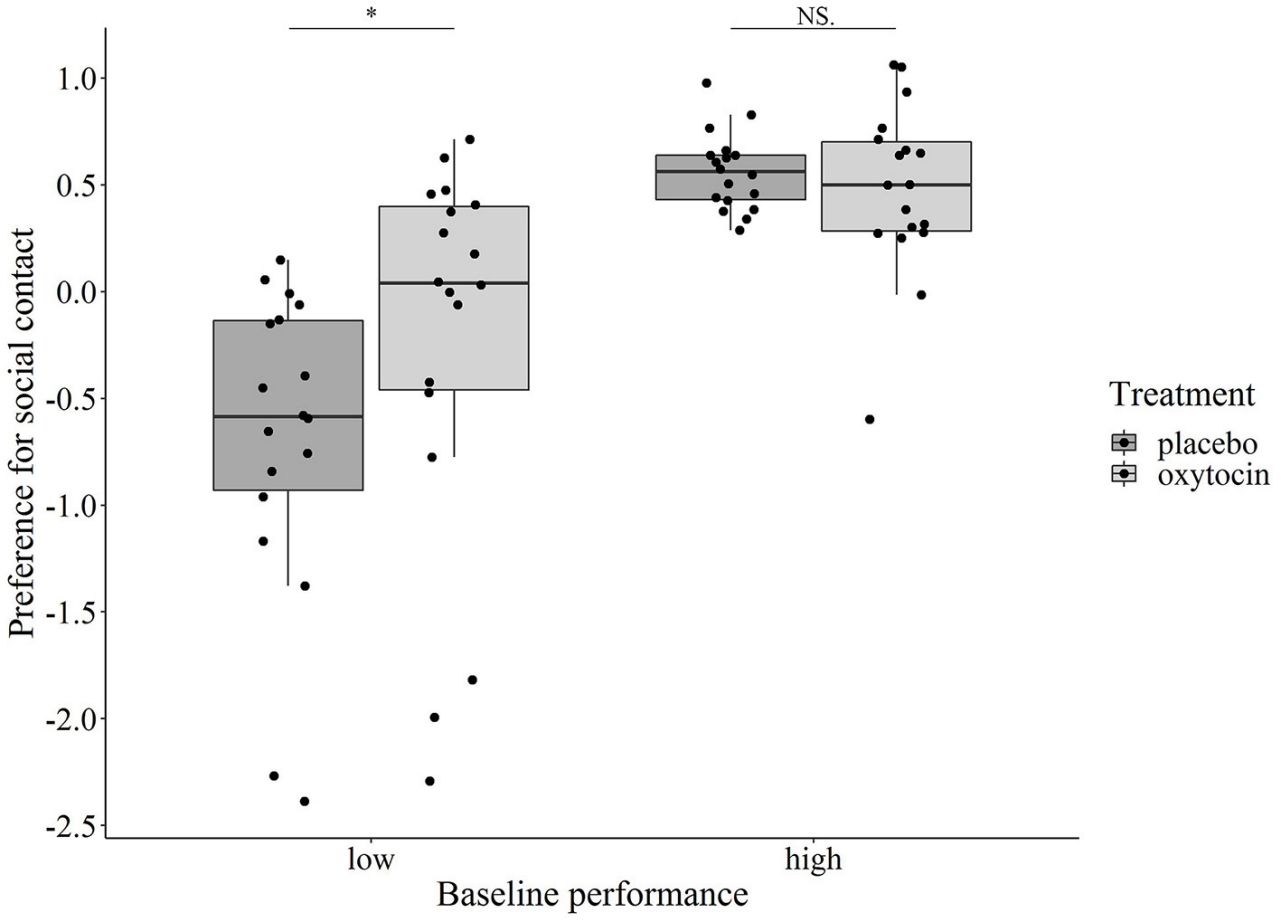
Relationship between the dogs' baseline performance and oxytocin/placebo treatment on their Preference for social contact component score. Oxytocin treatment significantly increased the component score in dogs with low baseline performance (^*^p = 0.004), while the effect of treatment was not significant in dogs with high baseline performance (*p* = 0.357).

Regarding the Preference for social contact component, *Sex* (male/female) and *Test session* (1st/2nd) had no main and interaction effects (*p* > 0.308 at removal); however, we found a significant interaction between *Treatment* and *Baseline performance* of the dogs (χ^2^ = 5.509, *p* = 0.019). When the *Baseline performance* was entered in the model as a categorical variable, its interaction with *Treatment* was still significant (χ^2^ = 10.389, *p* = 0.001). *Post-hoc* pairwise comparisons revealed that the effect of oxytocin administration was significant only in dogs with low baseline performance (Bonferroni-adjusted *p* = 0.004): these dogs showed a higher preference for social interaction with both familiar and unfamiliar humans in terms of contact seeking and response to human-initiated physical contact after intranasal administration of oxytocin than in the placebo condition. We found no effect of treatment in the case of dogs with high baseline performance (*p* = 0.357) (Figure 3).

Similar to the previous component, we found no main or interaction effect of *Sex* (male/female) or *Test session* (1st/2nd) on dogs' Non-aversive response to non-social threat (*p* > 0.156 at removal), but the *Treatment* × *Baseline performance* interaction was significant (χ^2^ = 7.647, *p* = 0.006). However, when we divided the dogs into low and high groups by the median, its interaction with *Treatment* was no longer significant (χ^2^ = 2.287, *p* = 0.130), and *post-hoc* comparisons also did not show a significant difference between the low and high groups in the effect of oxytocin vs. placebo treatment. When this interaction (and consequently, the categorical baseline performance variable) was removed from the model, the main effect of *Treatment* was not significant (χ^2^ = 0.013, *p* = 0.909).

**Figure 4:**
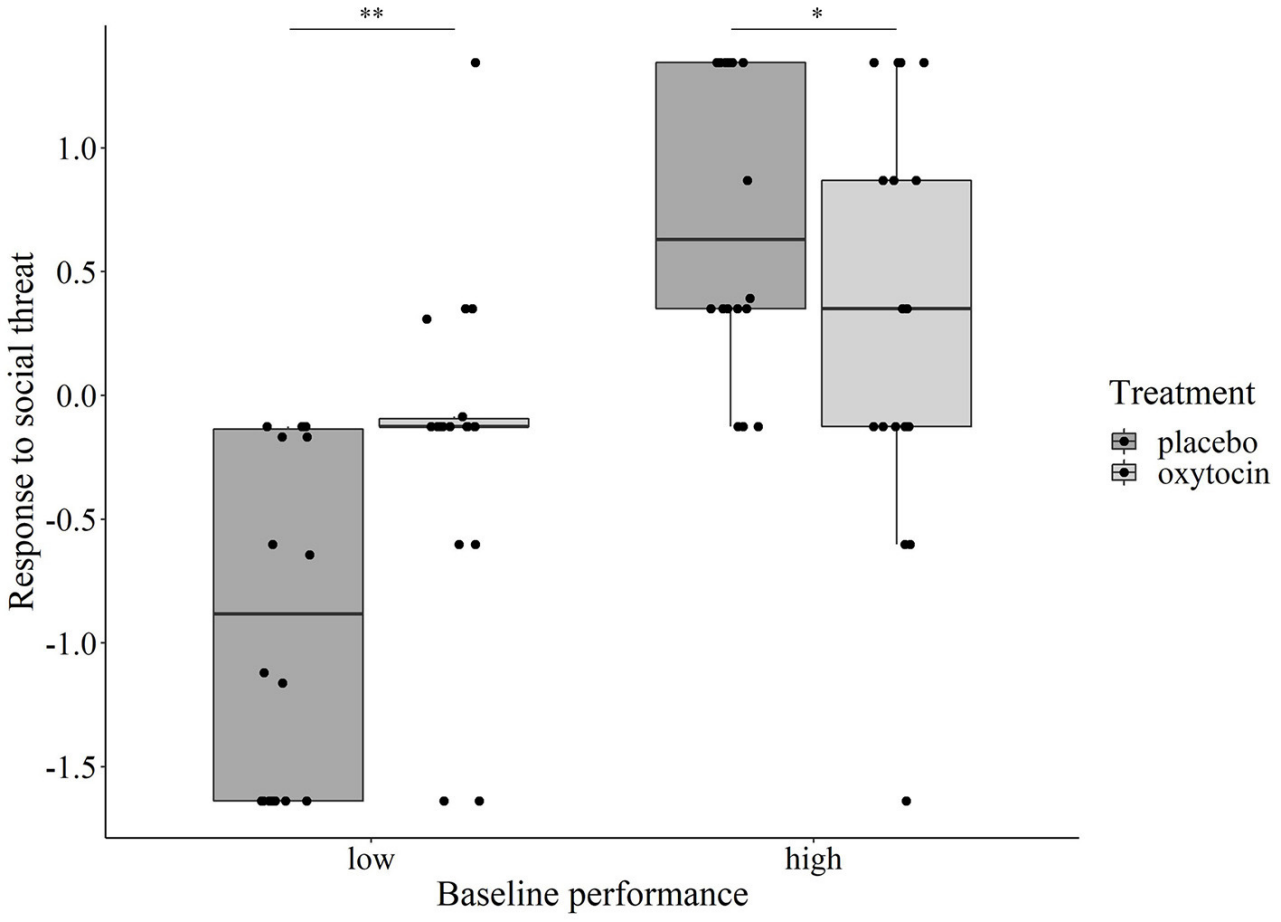
Relationship between the dogs' baseline performance and oxytocin/placebo treatment on their Non-aversive response to social threat component score. Oxytocin treatment significantly increased the component score in dogs with low baseline performance (^**^p = 0.002), while the effect of treatment was the opposite in dogs with high baseline performance (^*^p = 0.043).

Regarding the dogs' Non-aversive response to social threat, while *Test session* had no main or interaction effect (*p* > 0.374 at removal), we found two significant interactions: *Treatment* × *Sex* (χ^2^ = 3.901, *p* = 0.048) and *Treatment* × *Baseline performance* (χ^2^ = 34.559, *p* < 0.001). Regarding the former, *post-hoc* group comparisons did not show a significant or trend-level difference of the oxytocin treatment between males and females. However, for the *Treatment* × *Baseline performance* interaction, when the *Baseline performance* was entered in the model as a categorical variable, its interaction with *Treatment* was still significant (χ^2^ = 16.751, *p* < 0.001). *Post-hoc* tests revealed that oxytocin had an opposite effect on dogs' reaction to the experimenter's threatening approach depending on their baseline performance: dogs with low baseline performance showed significantly more positive reaction (i.e., tolerated the approach longer before moving away and/or were more likely to react with approach instead of avoidance) after receiving oxytocin as compared to receiving placebo (Bonferroni-adjusted *p* = 0.002), while for dogs with high baseline performance, intranasal administration of oxytocin led to a significantly less positive reaction (Bonferroni-adjusted *p* = 0.043) (Figure 4).

## Discussion

Although there is abundant research pointing to the social effects of oxytocin, less is known about how individual factors may moderate the effect of oxytocin in species other than humans. In this study, we set out to investigate the potential moderating effect of laboratory beagle dogs' baseline social competence on the association between oxytocin treatment and different aspects of social behavior. In accordance with our hypothesis and similar to human studies [e.g., Bartz et al. (12)], we have found that oxytocin treatment has a differential effect on the behavior, depending on the baseline social competence of the dogs. Individuals with low social competence benefit more from oxytocin treatment than socially more proficient individuals.

We tested various aspects of social behavior in laboratory beagles; however, three of the tasks included in the test battery (Training for eye contact, Directional gesturing, and Emotion recognition tasks) proved unsuitable for testing laboratory dogs. Even though these three tests aimed to measure phenotypes that, according to previous studies on family dogs, are affected by oxytocin [following gestural cues (30); willingness to make eye contact with humans (31); emotion recognition (32)], these test situations proved unfit for these purposes in our sample of laboratory beagles. This was probably because food rewards seemed insufficient to motivate the laboratory dogs to participate in the task, and/or their willingness to participate could have been influenced by a variety of situational factors, such as the unfamiliarity of the situation and/or the proximity of the stranger. The finding that one of the principal components, Willingness to interact, was composed of the dogs' willingness to accept food as well as behavioral variables coded in the abovementioned three tasks, clearly indicates that the degree of individual willingness to participate is one of the key challenges in testing laboratory dogs [similar to some other animals, like cats; e.g., Smith et al. (33)].

It is perhaps not surprising that the repetition of the test battery (the 1st and 2nd test sessions) had a significant effect on the Willingness to interact component—but only on this component. Dogs showed more willingness to actively participate in the test—regardless of the treatment—during the second test occasion. This suggests that they gathered positive experiences and/or habituated to the unfamiliarity of the situation enough to be able to focus on the task itself. Increasing food motivation probably also played an important role in this effect. Note that some of the dogs showed food neophobia during the first session (i.e., they were reluctant to consume the sausage probably because it was unfamiliar to them). During the repeated test session, however, all dogs that were willing to accept dry food readily accepted this new type of food reward.

The findings of the present study confirmed the differential effects of oxytocin treatment on the dogs depending on their baseline performance in the test battery (a measure of social competence). Although all four sets (components) of behavioral measures significantly interacted with treatment, these interaction effects were not uniformly strong in all principal components. Regarding the Willingness to interact and the Non-aversive reaction to non-social threat components, we found a significant treatment × baseline performance interaction only with a more sensitive assessment of the individual's baseline performance (i.e., when analyzed as a continuous score), whereas a comparison of the high- and low-performance groups (i.e., when grouped and analyzed as a categorical variable) did not show a significant difference between them. On the contrary, in the case of the other two components (Preference for social contact and Non-aversive reaction to social threat), significant differences remain between dogs even when categorized as high and low social competence groups in their reaction to oxytocin treatment. The results showed that oxytocin leads to a more positive reaction in both these components, but only in dogs with lower baseline performance. Regarding the Non-aversive reaction to social threat component, it is also interesting to note that oxytocin treatment led to a more negative (fearful) reaction in those dogs who showed a highly positive reaction to the threatening human in the placebo condition. This is in line with human studies suggesting that the effect of this neuropeptide depends on various contextual and inter-individual factors [for a review, see Bartz et al. (4)], and this phenomenon manifests itself also in the context-dependent influence of oxytocin on brain function (34). Results of another recent study indicate that oxytocin may serve as a warning system against potential threat cues in the environment and thus has the potential to facilitate active defensive behaviors (35). This finding contradicts the popular belief that oxytocin generally enhances social motivation and affiliative behaviors and further supports the results of human studies indicating that oxytocin can also induce antisocial effects, like aggression, envy, and distrust (8, 36), especially in negative situations like threat or competition (37).

Moreover, these results are also (partly) in agreement with the findings of Hernádi et al. (38). In their study, oxytocin treatment led to a less positive reaction to the threatening approach in dogs, compared to the placebo treatment, but only when the owner was performing the test, and no treatment effect was found when an unfamiliar experimenter approached the dog in a threatening manner. However, contrary to our results obtained on dogs with a low (fearful) baseline reaction, Hernádi et al. (38) did not find a positive effect of oxytocin, probably because their sample lacked dogs that showed severe fear in this situation (i.e., only 10% of their subjects responded with avoidance in the test); thus, a low number of dogs could positively benefit from the effect of oxytocin.

The current study has some limitations; firstly, there were a limited number of dogs available for analysis; secondly, we had no *a priori* information about behavior measures indicating a high (or low) level of social competence in dogs. Thus, the dogs' baseline performance was obtained by direct measurement of the individual dog's behavior in the different tests of the battery during the placebo condition. Moreover, the categorization of the dogs into high- and low-performance groups was rather rough. These could be especially problematic if task repetition affects the dogs' performance, as half of our subjects received a placebo in the second test session. Although we found such an effect only in the case of the Willingness to interact component, the repetition effect may overshadow a possible interaction between the baseline performance and the oxytocin treatment in this component. We advocate future studies to develop an independent measure of social competence that allows for a more objective method of categorization.

All in all, in at least two of the four components, we found that baseline performance indeed moderates the effect of oxytocin on the dogs' social behavior: oxytocin promotes increased social behavior only in individuals with poor social competence (low baseline performance in the placebo treatment) while having no or even a negative effect on dogs with sufficient social competence (high baseline performance). These results are consistent with the social salience hypothesis (4, 36, 37, 39), which suggests that oxytocin generally increases the salience of social cues, and its subsequent effects (whether positive or negative) depend on how these cues are interpreted, based on contextual information and the individuals' inclinations. Accordingly, oxytocin enhances positive behaviors (social approach, prosocial behaviors, and reduced stress) only when the attributed salience of the social context is positive, while in negative (competitive, aggressive, or threatening) contexts, oxytocin enhances negative behaviors (anxiety, competitive, or aggressive behaviors). In our case, increasing the salience of the social cues in positive contexts (Interaction with the caretaker and Greeting by an unfamiliar experimenter) indeed led to more positive reaction for dogs with low baseline preference for social contact (those that were less attuned to positive social information), while in the negative situation (Threatening approach), increasing the salience of the negative social cues led to more negative reactions for dogs with a more positive baseline reaction (those that were less attuned to negative social information). However, in the case of the Non-aversive reaction to social threat component, oxytocin also led to a more positive reaction for dogs with more negative baseline reactions, which suggests that other factors (e.g., social stress) may also play a role aside from the salience of social cues.

Alternatively, our findings are also in accordance with a putative inverted U-shaped correlation between endogenous oxytocin availability and social performance found in rats and humans [e.g., (40–42)]. Although endogenous oxytocin levels were not measured in our study, one might speculate that baseline performance was associated with endogenous oxytocin levels. Accepting these preconditions, administration of oxytocin to low performers could have increased the otherwise low baseline oxytocin level and shifted them into a concentration range more optimal for social functioning [for a similar finding in dogs, see Romero et al. (17)]. On the other hand, in the case of high baseline performers, an already high endogenous oxytocin level was further increased by external oxytocin administration, which could have pushed them out of the optimum concentration range, resulting in a less positive social behavior. A similar phenomenon has been described in Syrian hamsters, where at the baseline level, females were more socially competent than males, and the same dose of externally administered oxytocin reduced the reward value of social interaction in females while increasing it in males (43). Note that oxytocin, especially when present in high concentrations, can also bind to vasopressin receptors, and vasopressin can have opposite effects on behavior compared to oxytocin (44).

## Summary and Conclusions

In summary, the findings of the present study contribute to the growing body of evidence against the popular belief that oxytocin has a generally positive effect on social/prosocial behaviors and support the notion that oxytocin can help some individuals, but not others. This selective effect of oxytocin could explain at least some of the inconsistencies between previous dog oxytocin studies. Individuals with low social competence might be rare among family dogs, especially among pet dogs whose owners volunteer for research. Thus, different samples of family dogs could have different numbers of individuals or even no individuals on the negative end of the competence spectrum regarding the behavior in question, leading to a small or no association found in one study and significant associations in another. The social competence spectrum of laboratory dogs is supposedly much wider than that of family dogs, which made them a good choice for the purpose of our study. However, it is also reasonable to assume that for laboratory dogs on the negative end of the spectrum, even standard handling procedures can be stressful (21). In line with this notion, our findings show that oxytocin has a positive effect on the human-directed social behavior of these dogs, indicating that, in the absence of alternative opportunities (i.e., regular affiliative contact with humans), oxytocin administration has the potential to improve the wellbeing of these animals.

## Data Availability Statement

The raw data supporting the conclusions of this article will be made available by the authors, without undue reservation.

## Ethics Statement

The research was carried out in accordance with the Hungarian regulations on animal experimentation and the guidelines for the use of animals in research described by the Association for the Study of Animal Behavior (ASAB). Ethical approval was obtained from the National Animal Experimentation Ethics Committee (Ref. No. PE/EA/3742-4/2016). This study was also approved by the Local Ethical Committee of Gedeon Richter Plc. and was carried out in strict compliance with the European Directive 2010/63/EU regarding the care and use of laboratory animals for experimental procedures.

## Author Contributions

JT, VR, BT, and BL contributed to the conception and design of the study. JT was responsible for the funding acquisition. BT and EP carried out the behavioral experiments. BT performed the statistical analyses. BT and JT wrote the first draft of the manuscript. All authors contributed to manuscript revision and read and approved the submitted version.

## Funding

This research was supported by Gedeon Richter Plc. and has been implemented with the support provided by the Ministry of Innovation and Technology of Hungary from the National Research, Development and Innovation Fund, financed under the K128448 funding scheme.

## Conflict of Interest

VR, RK, GL, and BL are full-time employees of Gedeon Richter Plc. This study received funding from Gedeon Richter Plc. The funder had the following involvement with the study: Gedeon Richter Plc. provided laboratory space for the experiment and covered the costs associated with the care of laboratory beagles. The remaining authors declare that the research was conducted in the absence of any commercial or financial relationships that could be construed as a potential conflict of interest.

## Publisher's Note

All claims expressed in this article are solely those of the authors and do not necessarily represent those of their affiliated organizations, or those of the publisher, the editors and the reviewers. Any product that may be evaluated in this article, or claim that may be made by its manufacturer, is not guaranteed or endorsed by the publisher.
